# Isometric Strength Database for Muscle Maximal Voluntary Endurance Field Tests: Normative Data

**DOI:** 10.1186/s40798-021-00338-2

**Published:** 2021-07-12

**Authors:** Frédérick Janik, Claire Toulotte, Anne Laure Seichepine, Bernadette Masquelier, Fabienne Barbier, Claudine Fabre

**Affiliations:** 1 Univ. Lille, Univ. Artois, Univ. Littoral Côte d’Opale, ULR 7369 - URePSSS - Unité de Recherche Pluridisciplinaire Sport Santé Société, Lille, France; 2 Centre de Réadaptation Fonctionnelle “Les Hautois” - Groupe AHNAC, Oignies, France

**Keywords:** Normative database; Muscle prediction equation; Isometric field test; Outcome assessments; Reproducibility

## Abstract

**Background:**

Different field tests are used to evaluate muscle capacity, in particular maximal voluntary isometric endurance. However, although there are some normative values for a few muscle endurance tests, these do not consider the weight, height, gender, or age of individuals, which are well-known factors that influence muscle performance.

**Hypothesis/Purpose:**

The purpose of this study was to investigate the test–retest reproducibility of eight field tests and establish muscle endurance norms, in a healthy population, based on their anthropometric characteristics, which could allow the optimal evaluation of the entire muscle function in a quick manner.

**Design:**

Case series.

**Methods:**

This study was conducted in two phases. The first phase was to check the reproducibility inter- and intra-assessor for eight isometric muscle field tests on 20 volunteer subjects aged 40.9 ± 11.6 years old (age range, 21–58 years). The second part was to establish muscle maximal voluntary isometric endurance norms according to these tests on a total of 400 healthy participants grouped by age (50 males and females in each of the age brackets, 20–29; 30–39; 40–49; 50–59 years old, for a total of 200 males and 200 females).

**Results:**

The intra- and inter-assessor reproducibility tests are good for all muscle measurements (the intraclass correlation coefficients varied between 0.915 and 0.996 and the coefficient of variation between 3.6 and 11.8%). The area under the receiver operating characteristic curves demonstrates a good sensibility with values greater than 0.7 for each test. Each muscle belt presents same ratio regardless of the age and gender group. The simultaneous multiple regression analyses highlight that the anthropometric characteristics of subjects influence significantly the performance of isometric tests.

**Conclusion:**

This study has permitted establishing prediction equations in a healthy population according to their anthropometric characteristics as well as agonist/antagonist ratios for eight muscle isometric field tests after demonstrating a good reproducibility of all tests.

**Supplementary Information:**

The online version contains supplementary material available at 10.1186/s40798-021-00338-2.

## Key Points

- Predictive equations to establish muscle maximal voluntary isometric endurance present a good reproducibility for all muscle groups.
- These equations are useful in the field of prevention and/or rehabilitation (risk of falling, low back pain, etc.) or sport (muscle injury risks).

## Introduction

The assessment of muscle performance is a requirement in many fields such as prevention [1], sport [2], or rehabilitation [3], as the results of this assessment are used to prevent muscle injuries, guide and individualize training programs. Thus, the knowledge of reference values is a necessity to achieve the above goals. Muscle performance can be measured by different dynamometers, such as the isokinetic dynamometers [4], or the manual dynamometers [5]. Although the use of the isokinetic dynamometers is considered as the gold standard instrument to evaluate muscle strength [6], these devices remain very expensive, bulky, and require trained personnel to handle them and interpret the results. On the other hand, to measure muscle strength, the manual dynamometers present a good alternative, with a good reproducibility for the assessment of the upper and lower limbs [7, 8]. However, this reproducibility depends on the model of dynamometers used during the assessment [9]. In addition, although many authors propose reference values according to anthropometric data [5, 7, 10], these reference values depend on the model of the manual dynamometer and the calibration of the instrument [9]. So, the use of manual dynamometers remains limited as the comparison of the results between models is complicated.

Another way to simply evaluate muscle performance is by using field tests, which allows measuring maximal voluntary isometric endurance. In the literature, many field tests evaluating maximal voluntary isometric endurance have been validated, but, although some normative values exist for some muscle endurances tests [11, 12], these norms do not consider the weight, height, gender, and age of individuals. However, all these anthropometric data are well-known factors that positively or negatively influence muscle performance [13]. In addition, the postural position of some field tests often induces discomfort or pain [14] leading to an underestimation of muscle capacity because the test may be stopped prematurely. Therefore, it seems important to modify the postural positions of the subjects during field tests in order to decrease the limiting factors and to consider the anthropometric data of each subject while respecting the validity of the tests. Finally, the evaluation encountered in the literature on maximal voluntary isometric muscle endurance using isometric contractions is generally limited to four muscle groups: quadriceps, abdominals, lumbar, and quadratus lumborum muscles [12, 15]. Therefore, this assessment provides a somewhat limited overview of the muscle performance.

Another variable which must be taken into account in the evaluation of muscle performance is muscle imbalance or the agonist/antagonist muscle ratios. Indeed, in the case of muscle imbalance, a joint disorder and/or pain can result because the stabilization of the joint is no longer ensured [16]. With the knowledge of the muscle deficits, it is possible to propose an individualized training program that can restore a normal ratio [16]. Generally, to obtain the values of the muscle ratios, it is necessary to measure the strength of the agonist and antagonist muscles and then calculate their ratios. The most widely used method is the isokinetic method, but with the limitations of its use explained previously. McGill et al. (1999) have proposed an easier methodology than the isokinetic method, to determine the ratios in a healthy young adult population, using the endurance time of field tests [17]. However, in their study, muscle ratios were limited to three muscle groups, namely, the abdominals, lumbar, and quadratus lumborum muscles. In addition, all muscle ratios were determined from the performance of the lumbar muscles. Therefore, it is not possible to detect the specific imbalance in relationship with its joint and this form of assessment remains marginal. In addition, as highlighted by the authors, their ratios were established from a young population, which might not be applicable to an older population.

Therefore, we first hypothesize that it would be possible to validate field tests of maximal voluntary isometric muscle endurance with adaptive postural positions to best measure muscle performance; secondly, we hypothesize that the anthropometric data of the subjects could influence the maximal voluntary isometric muscle performance.

The objectives of this study were (1) to validate eight field tests of maximal voluntary isometric muscle endurance with adaptive postural positions; (2) to establish predictive equations of the entire muscle performance in a healthy population based on its anthropometric characteristics, which could allow the prevention of muscle injuries and/or to optimize the individualization of training programs in a quick and cheap manner.

## Materials and Methods

### Study Design

**Fig. 1:**
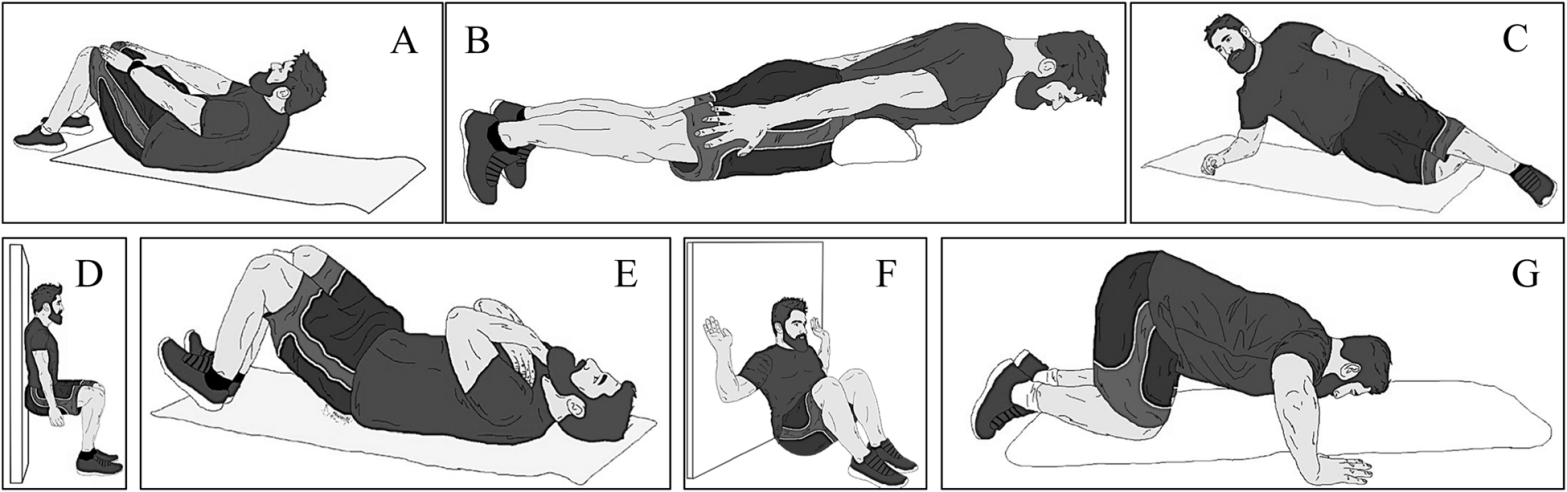
Figures illustrating the measurement muscle isometric endurance. (**A**) Trunk flexors, (**B**) trunk extensors, (**C**) quadratus lumborum muscles, (**D**) quadriceps muscles, (**E**) hamstrings muscles, (**F**) back muscles, and (**G**) chest muscles

This study was conducted in two phases. The first phase was to check the inter- and intra-assessor reproducibility for isometric muscle field tests, and the second part was to establish muscle maximal voluntary isometric endurance norms according to these tests. The isometric tests allow assessment of the trunk flexor, trunk extensor, quadratus lumborum, quadriceps, hamstring, and back and chest muscles (Fig. 1).

To be included in the study, the selection criteria for the two phases were as follows: no history of chronic or acute disease; a score < 9 on the Baecke questionnaire, which does not correspond to a sporting way of life [18]; no psychiatric or psychological disorders; and no contraindication for exercise.

Before being included in the study, the design, rules, and protocol were explained to each subject as required by the Declaration of Helsinki. Then each subject signed a written consent form. The study protocol was approved by the Behavioral Science Ethics Committee of University of Lille under No. 2019-380-S77.

### Part 1: Reproducibility of Muscle Maximal Voluntary Isometric Endurance Field Tests

The aim of this first part of the study was to examine intra- and inter-assessor reproducibility in a test–retest during isometric field tests in order to assess the maximal voluntary isometric endurance of the quadriceps, hamstring, abdominal, trunk extensor, right and left quadratus lumborum, and back and chest muscles (Fig. 1).

#### Subjects

Twenty subjects (9 males, 11 females), with a mean age of 40.9 ± 11.6 years (mean ± SD), were included (Table 1). Subjects completed the Baecke questionnaire [18] to determine their physical activity level and were asked about any history of medical conditions in order to verify the entry/exclusion criteria.

**Table 1:** Characteristics of the study population

	Test reproducibility	Muscle norms participants		
	Participants (*n = 20*)	Males (*n = 200*)	Females (*n = 200*)	*p value*
	Mean ± SD	Mean ± SD	Mean ± SD	
Age (years)	40.9 ± 11.6	39.4 ± 11.4	39.7 ± 11.5	NS
Weight (kg)	79.9 ± 16.8	82.9 ± 13.7	66.3 ± 14.6	***
Height (cm)	172.6 ± 7.9	178.0 ± 7.5	165.8 ± 5.9	***
BMI (kg/m^2^)	26.7 ± 4.3	26.2 ± 4.3	24.1 ± 5.0	NS
Baecke score (a.u)	7.1 ± 1.1	7.9 ± 2.1	7.5 ± 1.8	NS
Trunk flexor muscles endurance (s)	147.3 ± 77.8	121.7 ± 47.9	120.0 ± 67.8	NS
Back extensor muscles endurance (s)	210.0 ± 76.8	181.0 ± 66.8	183.2 ± 92.8	NS
Right quadratus lumborum muscles endurance (s)	80.5 ± 35.7	76.9 ± 34.7	76.7 ± 35.2	NS
Left quadratus lumborum muscles endurance (s)	81.9 ± 30.3	77.7 ± 33.1	77.4 ± 32.8	NS
Quadriceps muscles endurance (s)	75.3 ± 28.4	81.7 ± 29.8	76.8 ± 32.4	NS
Hamstrings muscles endurance (s)	243.2 ± 110.3	240.2 ± 76.5	224.2 ± 90.2	NS
Back muscles endurance (s)	185.7 ± 77.4	183.7 ± 58.8	186.3 ± 79.8	NS
Chest muscles endurance (s)	76.3 ± 25.5	64.3 ± 19.4	63.7 ± 28.8	NS

The mean and standard deviation of all endurance muscles tests for test reproducibility correspond to overall mean of the last three assessments “A2,” “B1,” and “B2;” *BMI* body mass index, *a.u* arbitrary units, *NS* non-significant; ***p < 0.001; *A2* second assessment of assessor A, *B1* first assessment of assessor B, *B2* second assessment of assessor B

#### Protocol

Two assessors, A and B, each performed two evaluation sessions (A1 and A2 for assessor A and B1 and B2 for assessor B). In order to avoid habituation effect, data from A- and B-rater were collected randomly. Prior to data collection, the assessors practised using the test protocols to ensure that standardized procedures were employed.

Each evaluation session lasted for 2 weeks with a break of at least 24 h between each one. This 24-h interval was chosen in order to avoid the impact of fatigue resulting from two sessions being too close together [19, 20]. Only the data of the last three evaluation sessions (A2, B1, and B2) were analyzed, with the first session (A1) serving as a familiarization and therefore the results of this session were not considered in the analysis of the data.

No attempt was made to standardize the order in which, or the time of day when, the evaluations were completed. Measurements from the two assessors were recorded on separate data collection forms to ensure that they were blinded to each other’s results and their own previous results. Both assessors carried out two assessment sessions for each subject and applied each field test in random order. The randomization was performed with the R software by assigning a number to each test. During the assessment, the antagonist muscle was tested after the agonist muscle. Prior to the assessment of each test, the assessors explained and demonstrated the test procedure to the subject.

Each assessment session started with 10 min of cardiorespiratory warm-up on a cycloergometer at 65% of the target heart rate, as determined by the Karvonen formula [21], and subjects benefited from at least 5-min rest between each test.

#### Guidelines for Postures During the Tests

For this study, the objective was to reduce pain generated by the discomfort of the position tests during the evaluation. To do this, the postural position of some tests was modified (Fig. 1). These modifications and all instructions for all tests, including pretest cueing, starting position, subject instructions, and termination criteria, are presented in the Additional file 1: Appendix. Next, there followed a brief presentation of the used tests with their alterations or not: (1) trunk flexor muscles were evaluated with the test of Ito et al. [14]. Some modifications were carried out, including the position of the legs and the termination criteria; (2) trunk extension was evaluated using the test of Ito et al. [14] without modification; (3) right and left quadratus lumborum muscles were evaluated using a modification of the positions in McGill et al.’s tests [17]. The modification was the position of the legs; (4) the isometric test of quadriceps using the original Killy test described by Bernard et al. [22] was used; (5) for the hamstring muscles test, a derivative of the bridge exercise to the neutral spine alignment position described by Youdas et al. [23] was used. These modifications included the pelvis position and the feet position; (6) the back muscles test was an adaptation of the behind-the-neck lat pull-down described by Sperandei et al. [24]. This test required the same movement as the behind-the-neck lat pull-down but the subject sat on the floor, with their back, shoulders, elbows, hands, and head against the wall. In addition, this test was carried out without equipment and in an isometric way; (7) for the chest muscles test, a derivative of the push-up on the knees exercise described by Vossen et al. [25] was used. In our study, this test was performed in an isometric way, so the termination criteria were not the same.

Participants were individually instructed and supervised by experienced therapists specifically trained in the testing methodology. In each of these tests, the weight represented the load.

### Part 2: Creation of Muscle Norms

#### Subjects

Four hundred healthy Caucasian participants segmented by age (50 males and 50 females in each age bracket, 20–29 years old; 30–39 years old; 40–49 years old; and 50–59 years old; a total of 200 males and 200 females) were included. The female group was aged 39.7 ± 11.5 years and the male group was aged 39.4 ± 11.4 years (Table 1).

#### Protocol

Prior to isometrics testing, participants performed 10 min of warm-up, including all articulations of the body. After that, all subjects performed the eight maximal voluntary isometric muscle endurance tests. Each test was carried out once per session. Tests were conducted in random order with at least 5-min rest between two tests. As for the first part, randomization was performed using the R software by assigning a number to each test. The antagonist muscles were systematically tested after the agonist muscles.

Prior to the performance of each test, the assessor explained and demonstrated the test to subjects using standardized instructions. For this part, the same researcher conducted all the tests.

#### Instructions for End of Tests Parts 1 and 2

For all endurance tests, in both parts 1 and 2, subjects were encouraged to hold the test position until exhaustion, and were given feedback if they deviated from the position. Tests were terminated when the subject could not maintain the position or if there were any obvious signs of fatigue (not maintaining the position in spite of verbal feedback, for example) or a significant emergence of pain or other symptoms. The maximum holding time was recorded in seconds using a stopwatch. The stopwatch was triggered when the subject was in the right position.

### Statistical Analysis

#### Intra- and Inter-assessor Reproducibility

Intra- and inter-assessor reproducibility was estimated using intraclass correlation coefficients (ICCs). The intra-assessor reproducibility was quantified by calculating the ICC between the measurement conducted by the same assessor “B1” and “B2.” The inter-assessor reproducibility was measured by calculating the ICC between the measurement of assessor A (“A2”) and assessor B (“B1” and “B2”). For each ICC, error range and repeatability were calculated with standard error of the measurement (SEM), 95% confidence intervals (CIs), and 95% limits of agreement (LOAs). The standard error of the measurement (SEM) was calculated according to the formula SEM = SD √(1-ICC) [26], to provide an estimate of the precision of measurement, expressed in the units of the measure. The SEM was divided by the mean of the two measurements and multiplied by 100 to give a percentage value (SEM%) [27]. A percentage of 95% of LOA demonstrates the range of measurement error within the sample [28]. The interpretation of the ICCs was obtained according to the study by Shrout: reproducibility was considered strong if the ICC was between 1 and 0.81, moderate between 0.80 and 0.61, fair between 0.60 and 0.41, low between 0.40 and 0.11, and non-reproducible if less than 0.10 [29].

In order to determine the absolute reproducibility, the coefficient of variation (CV) was calculated. The CV is expressed as a percentage (CV%) and is calculated by dividing the standard deviation by the mean, multiplied by 100, for each test. The CV values of 10% [30] and 15% [31] have been used to consider the level of absolute reproducibility of the measurement (CV < 15% = good reproducibility; CV < 10% = excellent reproducibility).

The Breusch-Pagan test was used to verify the heteroscedasticity in the regression model. The heteroscedasticity of the model was confirmed when p value < 0.05 [32]. The performances in all isometric tests for each session of evaluation were expressed as mean ± standard deviation (mean ± SD). Statistical analysis was performed using the SPSS version 20.0 software and the Breusch-Pagan test was performed using the R software version 4.0.4.

#### Muscle Norms

A sample size of 34 participants per group was needed to detect a 10% difference (power of 0.9 and *p* < 0.05) according to the results of the studies by Claxton et al. (2009) [11] and Evans et al. (2007) [12]. However, the present data were part of a larger study in which more variables were assessed, which required a larger sample size. So, 400 subjects were included in this study.

The sensitivity of all tests was determined by a receiver operating characteristic (ROC) analysis, as a function of the age brackets 20–29 years and 50–59 years for male and female groups. The area under the ROC curve (AUC) is widely used to estimate the predictive accuracy of distributional models derived from presence/absence data [33]. The relationship between AUC and the sensitivity was considered excellent between 1 and 0.9, very good between 0.9 and 0.8, good between 0.8 and 0.7, average between 0.7 and 0.6, and poor between 0.6 and 0.5 [34].

The gender influence was evaluated by one-way ANOVA. To establish the norms for the eight muscle field tests, multiple regression analyses were conducted to determine the influence of the anthropometric data (age, weight, and height) on the performance of isometric tests. All values were expressed as mean ± standard deviation (mean ± SD). For the gender parameter, data normality was tested using the Shapiro–Wilk test. The significance level was set at the 0.05 level. The statistical analyses were performed using the R software version 3.5.0.

## Results

Anthropometric characteristics of all subjects included in this study and the overall means of the last three assessments “A2,” “B1,” and “B2” for all the isometric tests are presented in Table 1.

### Part 1: Reproducibility of Muscle Endurance Tests

Table 2 presents the results of the intra-assessor (“B1” and “B2”) reproducibility tests for all muscle measurements. The ICC values of the muscle endurance measurements vary between 0.946 and 0.989, thereby indicating excellent reproducibility. The bias values range from −3.9 to 1.0, staying close to zero and indicating a slight systematic improvement between measurements.

**Table 2:** Intra-assessor intraclass correlation coefficients

Muscle isometric tests	B1 (sec.) *Mean ± SD*	B2 (sec.) *Mean ± SD*	Bias (sec.) *Mean ± SD*	CV (%)	ICC [95% CI]	95% LOA (sec.)		SEM (sec.)	SEM (%)
						Lower	Upper		
Trunk flexor muscles	145.2 ± 79.3	146.0 ± 79.1	−0.8 ± 20.1	9.1	0.984 [0.961‑0.994]	−40.15	38.65	2.54	1.75
Back extensor muscles	207.4 ± 81.9	210.9 ± 71.9	−3.5 ± 26.9	8.9	0.970 [0.924‑0.988]	−56.27	49.27	4.66	2.23
Right quadratus lumborum muscles	77.5 ± 36.0	81.4 ± 36.4	−3.9 ± 14.7	11.3	0.956 [0.891‑0.983]	−32.71	24.91	3.08	3.88
Left quadratus lumborum muscles	79.2 ± 32.5	82.8 ± 30.6	−3.6 ± 12.9	11.1	0.955 [0.889‑0.982]	−28.85	21.65	2.73	3.37
Quadriceps muscles	74.1 ± 27.8	74.5 ± 31.2	−0.4 ± 11.9	10.3	0.959 [0.897‑0.984]	−23.84	22.94	2.42	3.25
Hamstrings muscles	241.8 ± 114.3	242.2 ± 112.6	−0.5 ± 24.3	6.6	0.989 [0.972‑0.996]	−48.11	47.21	2.55	1.05
Back muscles	185.8 ± 80.6	184.8 ± 75.5	1.0 ± 19.0	6.9	0.986 [0.964‑0.994]	−36.18	38.18	2.24	1.21
Chest muscles	74.2 ± 26.2	77.1 ± 27.6	−2.9 ± 12.1	10.5	0.946 [0.866‑0.978]	−26.69	20.89	2.82	3.73

*CV* coefficient of variation, *ICC* intraclass correlation coefficients, *CI* confidence intervals, *SEM* standard error of measurement, *LOA* limits of agreement, *sec*. seconds, *%* percentage, *SD* standard deviation, *B1* first assessment of assessor B, *B2* second assessment of assessor B

Tables 3 and 4 present the results of the inter-assessor (between assessments of “B1” and “A2” and between assessments of “B2” and “A2”) reproducibility tests for all muscle measurements. The ICC values indicate excellent inter-assessor reproducibility, with measurements varying between 0.915 and 0.996 for the measures of “B1” and “A2” assessments and between 0.955 and 0.996 for the measures of “B2” and “A2” assessments. For inter-assessor comparison, the bias values are negative (ranging from −5.6 to −0.8 for the measurement between “B1” and “A2” and from −4.9 to −0.6 for the measurement between “B2” and “A2”), indicating a slight systematic improvement between measurements.

**Table 3:** Inter-assessor intraclass correlation coefficients between assessments “B1” and “A2”

Muscle isometric tests	B1 (sec.) *Mean ± SD*	A2 (sec.) *Mean ± SD*	Bias (sec.) *Mean ± SD*	CV (%)	ICC [95% CI]	95% LOA (sec.)		SEM (sec.)	SEM (%)
						Lower	Upper		
Trunk flexor muscles	145.2 ± 79.3	150.8 ± 78.9	−5.6 ± 10.2	5.1	0.995 [0.983‑0.998]	−25.66	14.46	0.72	0.50
Back extensor muscles	207.4 ± 81.9	211.8 ± 80.2	−4.5 ± 13.0	4.5	0.993 [0.982‑0.997]	−30.01	21.11	1.09	0.52
Right quadratus lumborum muscles	77.5 ± 36.0	82.6 ± 36.6	−5.1 ± 9.1	9.3	0.980 [0.937‑0.993]	−22.80	12.70	1.28	1.61
Left quadratus lumborum muscles	79.2 ± 32.5	83.6 ± 29.2	−4.4 ± 10.1	8.6	0.969 [0.916‑0.988]	−24.29	15.49	1.79	2.21
Quadriceps muscles	74.1 ± 27.8	77.3 ± 27.3	−3.2 ± 9.7	8.7	0.966 [0.914‑0.987]	−22.27	15.87	1.79	2.42
Hamstrings muscles	241.8 ± 114.3	245.6 ± 109.7	−3.9 ± 14.5	4.6	0.996 [0.989‑0.998]	−32.21	24.51	0.91	0.38
Back muscles	185.8 ± 80.6	186.6 ± 80.1	−0.8 ± 15.7	5.3	0.991 [0.977‑0.996]	−31.51	29.91	1.49	0.80
Chest muscles	74.2 ± 26.2	77.7 ± 23.7	−3.5 ± 13.9	11.8	0.915 [0.788‑0.966]	−30.81	23.81	4.06	5.37

*CV* coefficient of variation, *ICC* intraclass correlation coefficients, *CI* confidence intervals, *SEM* standard error of measurement, *LOA* limits of agreement, *sec.* seconds, *%* percentage, *SD* standard deviation, *B1* first assessment of assessor B, *A2* second assessment of assessor A

**Table 4:** Inter-assessor intraclass correlation coefficients between assessments “B2” and “A2”

Muscle isometric tests	B2 (sec.) *Mean ± SD*	A2 (sec.) *Mean ± SD*	Bias (sec.) *Mean ± SD*	CV (%)	ICC [95% CI]	95% LOA (sec.)		SEM (sec.)	SEM (%)
						Lower	Upper		
Trunk flexor muscles	146.0 ± 79.1	150.8 ± 78.9	−4.9 ± 13.8	5.8	0.992 [0.979‑0.997]	−31.93	22.23	1.24	0.85
Back extensor muscles	210.9 ± 71.9	211.8 ± 80.2	−1.0 ± 20.2	6.4	0.983 [0.957‑0.993]	−40.58	38.68	2.64	1.26
Right quadratus lumborum muscles	81.4 ± 36.4	82.6 ± 36.6	−1.2 ± 11.7	8.5	0.975 [0.936‑0.990]	−24.08	21.78	1.85	2.33
Left quadratus lumborum muscles	82.8 ± 30.6	83.6 ± 29.2	−0.8 ± 12.7	8.1	0.955 [0.886‑0.982]	−25.71	24.11	2.70	3.33
Quadriceps muscles	74.5 ± 31.2	77.3 ± 27.3	−2.8 ± 11.9	9.2	0.957 [0.893‑0.983]	−26.06	20.56	2.47	3.32
Hamstrings muscles	242.2 ± 112.6	245.6 ± 109.7	−3.4 ± 13.6	3.6	0.996 [0.991‑0.998]	−30.00	23.20	0.86	0.35
Back muscles	184.8 ± 75.5	186.6 ± 80.1	−1.8 ± 14.6	4.5	0.991 [0.978‑0.997]	−30.47	26.87	1.39	0.75
Chest muscles	77.1 ± 27.6	77.7 ± 23.7	−0.6 ± 10.8	8.4	0.956 [0.889‑0.983]	−21.69	20.49	2.26	3.00

*CV* coefficient of variation, *ICC* intraclass correlation coefficients, *CI* confidence intervals, *SEM* standard error of measurement, *LOA* limits of agreement; *sec.* seconds, *%* percentage, *SD* standard deviation, *B2* second assessment of assessor B, *A2* second assessment of assessor A

Regarding the absolute reproducibility, the CV values vary between 3.6 and 11.8% (Tables 2, 3, 4). The intra-assessor (Table 2) absolute reproducibility is between good and excellent reproducibility for all tests (minimum measurement, 6.6% for the hamstring test; maximum measurement, 11.3% for the right quadratus lumborum test). The absolute reproducibility of inter-assessors (Tables 3 and 4) is also between good and excellent for all tests (minimum measurement, 3.6% for the hamstring test between “B2” and “A2;” maximum measurement, 11.8% for the chest muscle test between “B1” and “A2”).

With regard to absolute reproducibility, the values of SEM% demonstrate a good reproducibility of the accuracy and precision of the measured values. Indeed, the SEM values of intra- or inter-assessor measurements are less than 10% of the average measured value, so the measurement errors are small, and therefore the measurement is reliable [35].

The Breusch-Pagan p values for all tests are greater than 0.05 (the lowest value, BP = 3.6, df = 1, p value = 0.07 for the chest muscle tests between “B1” and “A2”); thus, the null hypotheses is accepted, i.e., the data are homoscedastic.

The intra-assessor and inter-assessor coefficient of variation (CV) was also used to measure the absolute reproducibility of agonist/antagonist ratios (Table 5). Muscle ratios were determined by dividing the holding time of the anterior chain muscles by the holding times of the posterior chain muscles for all muscle belts. For each muscle belt, the CV values vary between 5.8 and 12.9%, corresponding to between good to excellent reproducibility for all agonist/antagonist ratios.

**Table 5:** Intra-assessor and inter-assessor coefficient of variation for the muscle agonist/antagonist ratios

Agonist/antagonist ratios	CV (%) Intra-assessor	CV (%) Inter-assessor (B1-A2)	CV (%) Inter-assessor (B2-A2)
Trunk flexor/back extensor	12.8	8.7	5.8
Left/right quadratus lumborum	10.6	6.4	10.7
Quadriceps/hamstrings	12.5	8.1	10.1
Chest muscles/back muscles	11.6	12.9	10.6

*CV* coefficient of variation, *B1* first assessment of assessor B, *B2* second assessment of assessor B, *A2* second assessment of assessor A

### Part 2: Muscle Norms

**Fig. 2:**
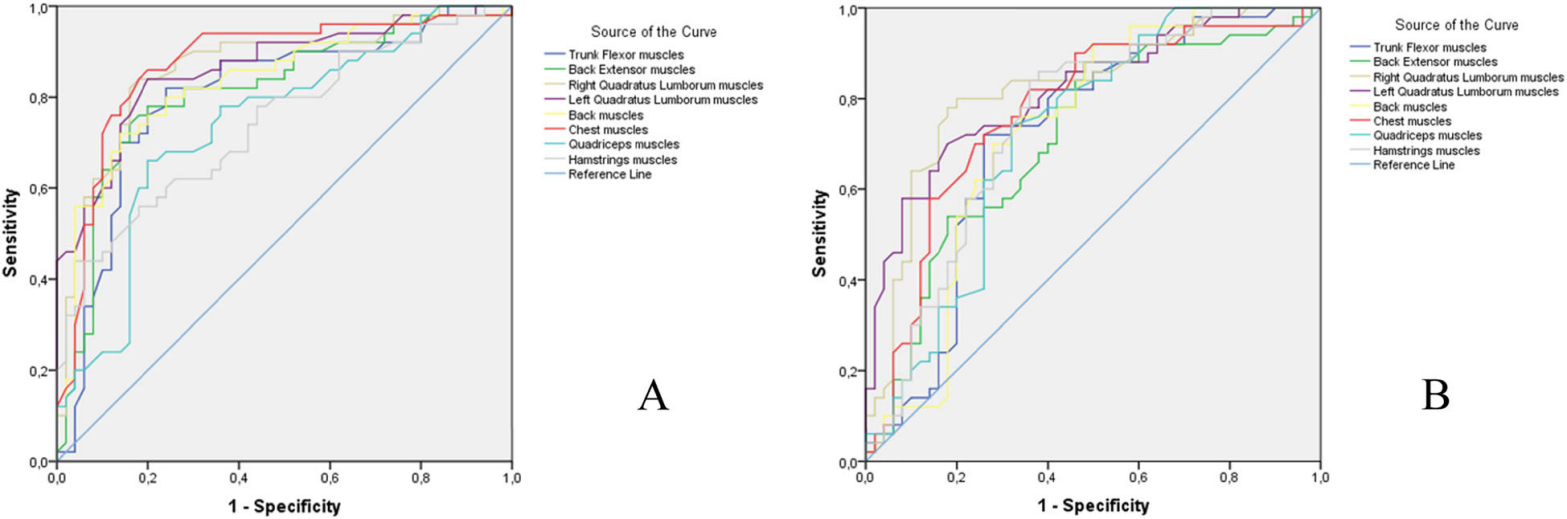
ROC curves for all tests as a function of the age brackets. (**A**) Male group; (**B**) female group

The ROC curves are presented in Fig. 2. The AUC demonstrates a good sensibility according to age brackets with values greater than 0.7 for each test (Tables 6 and 7).

**Table 6:** Receiver operating characteristic (ROC) analysis for all tests as a function of the age brackets for male group

	20‑29 (*n* = 50)	50‑59 (*n* = 50)	*p*	ROC		
	*Mean ± SD*	*Mean ± SD*		AUC	95% CI	*P*
Trunk flexor muscles	146.4 ± 41.5***	97.7 ± 41.5	< 0.001	0.803	0.712‑0.894	< 0.001
Back extensor muscles	216.8 ± 59.7***	144.7 ± 56.3	< 0.001	0.811	0.723‑0.900	< 0.001
Right quadratus lumborum muscles	98.9 ± 33.2***	55.5 ± 24.2	< 0.001	0.864	0.790‑0.939	< 0.001
Left quadratus lumborum muscles	100.3 ± 32.2***	56.7 ± 22.5	< 0.001	0.864	0.792‑0.936	< 0.001
Quadriceps muscles	93.6 ± 28.7***	69.0 ± 26.9	< 0.001	0.739	0.640‑0.837	< 0.001
Hamstrings muscles	274.1 ± 80.1***	204.9 ± 65.9	< 0.001	0.742	0.646‑0.838	< 0.001
Back muscles	212.9 ± 56.0***	143.1 ± 44.9	< 0.001	0.835	0.755‑0.916	< 0.001
Chest muscles	75.2 ± 17.5***	50.2 ± 14.7	< 0.001	0.872	0.798‑0.947	< 0.001

*ROC* receiver operating characteristic, *AUC* area under the curve, *CI* confidence intervals, *SD* standard deviation; ***p < 0.001

**Table 7:** Receiver operating characteristic (ROC) analysis for all tests as a function of the age brackets for female group

	20‑29 (*n* = 50)	50‑59 (*n* = 50)	*p*	ROC		
	*Mean ± SD*	*Mean ± SD*		AUC	95% CI	*P*
Trunk flexor muscles	140.6 ± 63.1**	97.2 ± 66.6	0.001	0.719	0.615‑0.823	< 0.001
Back extensor muscles	212.6 ± 92.9**	154.7 ± 95.9	0.002	0.712	0.610‑0.815	< 0.001
Right quadratus lumborum muscles	96.6 ± 36.6***	55.5 ± 30.1	< 0.001	0.818	0.733‑0.904	< 0.001
Left quadratus lumborum muscles	96.5 ± 33.9***	57.2 ± 27.7	< 0.001	0.816	0.733‑0.898	< 0.001
Quadriceps muscles	88.74 ± 29.7***	64.8 ± 32.2	< 0.001	0.722	0.620‑0.824	< 0.001
Hamstrings muscles	261.4 ± 84.0***	189.3 ± 92.8	< 0.001	0.749	0.651‑0.847	< 0.001
Back muscles	212.5 ± 65.6***	155.8 ± 84.7	< 0.001	0.730	0.627‑0.832	< 0.001
Chest muscles	74.5 ± 27.2***	49.8 ± 25.9	< 0.001	0.772	0.677‑0.867	< 0.001

*ROC* receiver operating characteristic, *AUC* area under the curve, *CI* confidence intervals, *SD* standard deviation; **p < 0.01; *** *p* < 0.001

For the male and female groups, the average hold time for each test is presented in Table 1. Each test carried out was stopped following muscle fatigue by the participant themself or by the assessor when the participant showed obvious signs of fatigue (maintaining of the initial position was impossible). No test was stopped following an emergence of pain or other symptoms.

Prediction equations to estimate muscle performance according to anthropometric criteria are presented in Tables 8 and 9. The muscle ratios of each muscle belt present no significant difference between men and women and no difference according to age bracket (Table 10).

**Table 8:** Male performance prediction equations for all isometric tests

Muscle endurance tests	Predictive Equations	*R*^*2*^
Trunk flexor muscles	−10.92 − 1.18 × (age; years)^***^ − 0.87 × (weight; kg)^***^ + 1.41 × (height; cm)^**^	0.200
Back extensor muscles	112.42 − 1.89 × (age; years)^***^ − 1.10 × (weight; kg)^**^ + 1.32 × (height; cm)^*^	0.195
Right quadratus lumborum muscles	17.44 − 1.13 × (age; years)^***^ − 0.78 × (weight; kg)^***^ + 0.94 × (height; cm)^**^	0.294
Left quadratus lumborum muscles	35.49 − 1.11 × (age; years)^***^ − 0.87 × (weight; kg) ^***^ + 0.89 × (height; cm)^**^	0.342
Quadriceps muscles	−9.59 − 0.60 × (age; years)^***^ − 0.49 × (weight; kg)^**^ + 0.87 × (height; cm)^**^	0.157
Hamstrings muscles	35.17 − 1.66 × (age; years)^***^ − 1.33 × (weight; kg)^***^ + 2.14 × (height; cm)^**^	0.168
Back muscles	125.19 − 1.94 × (age; years)^***^ − 0.84 × (weight; kg)^**^ + 1.15 × (height; cm)^*^	0.225
Chest muscles	38.76 − 0.64 × (age; years)^***^ − 0.41 × (weight; kg)^***^ + 0.48 × (height; cm)^**^	0.284

*kg* kilograms, *cm* centimeter; *p < 0.05; **p < 0.01; ***p < 0.001

Interpretation. The expected holding time for maximal endurance trunk flexor test for a 51-year man with height of 184 cm and a weight of 97 kg is: −10.92 − (1.18 × 51) − (0.87 × 97) + (1.41 × 184) = 103.95 s.

**Table 9:** Female performance prediction equations for all isometric tests

Muscle endurance tests	Performance prediction equations	*R*^*2*^
Trunk flexor muscles	−355.82 − 0.96 × (age; years)^*^ − 1.69 × (weight; kg)^***^ + 3.78 × (height; cm)^***^	0.225
Back extensor muscles	−683.83 − 1.30 × (age; years)^*^ − 2.20 × (weight; kg)^***^ + 6.42 × (height; cm)^***^A.	0.258
Right quadratus lumborum muscles	−40.55 − 1.09 × (age; years)^***^ − 0.92 × (weight; kg)^***^ + 1.34 × (height; cm)^***^	0.331
Left quadratus lumborum muscles	−29.35 − 1.06 × (age; years)^***^ − 0.88 × (weight; kg)^***^ + 1.25 × (height; cm)^***^	0.353
Quadriceps muscles	−257.23 − 1.74 × (age; years)^***^ − 1.47 × (weight; kg)^***^ + 3.68 × (height; cm)^***^	0.196
Hamstrings muscles	−57.63 − 0.64 × (age; years)^***^ − 0.45 × (weight; kg)^**^ + 1.06 × (height; cm)^**^	0.217
Back muscles	−81.78 − 0.56 × (age; years)^**^ − 0.74 × (weight; kg)^***^ + 1.39 × (height; cm)^***^	0.201
Chest muscles	−282.91 − 1.92 × (age; years)^***^ − 1.88 × (weight; kg)^***^ + 4.27 × (height; cm)^***^	0.164

*kg* kilograms, *cm* centimeter; *p < 0.05; **p < 0.01; ***p < 0.001

Interpretation. The expected holding time for maximal endurance trunk flexor test for a 34-year-old female with height of 173 cm and a weight of 56 kg is: −355.82 − (0.96 × 34) − (1.69 × 56) + (3.78 × 173) = 170.84 s

**Table 10:** Variation of agonist/antagonist ratios according to age brackets

Agonist/antagonist ratios	Males					Females				
	[20‑29] *n* = 50	[30‑39] *n* = 50	[40‑49] *n* = 50	[50‑59] *n* = 50	Global *n* = 200	[20‑29] *n* = 50	[30‑39] *n* = 50	[40‑49] *n* = 50	[50‑59] *n* = 50	Global *n* = 200
	Mean ± SD	Mean ± SD	Mean ± SD	Mean ± SD	Mean ± SD	Mean ± SD	Mean ± SD	Mean ± SD	Mean ± SD	Mean ± SD
Trunk flexor/back extensor	0.70 ± 0.21	0.70 ± 0.25	0.70 ± 0.20	0.70 ± 0.22	0.,70 ± 0.22	0.70 ± 0.23	0.70 ± 0.32	0.70 ± 0.19	0.70 ± 0.46	0.,70 ± 0.32
Left/right quadratus lumborum	1.00 ± 0.14	1.00 ± 0.19	1.00 ± 0.19	1.00 ± 0.24	1.,00 ± 0.19	1.00 ± 0.17	1.00 ± 0.17	1.00 ± 0.18	1.00 ± 0.35	1.,00 ± 0.23
Quadriceps/hamstrings	0.37 ± 0.09	0.37 ± 0.12	0.37 ± 0.12	0.37 ± 0.13	0.,37 ± 0.12	0.37 ± 0.14	0.37 ± 0.18	0.37 ± 0.12	0.37 ± 0.17	0.37 ± 0.16
Chest muscles/back muscles	0.36 ± 0.13	0.36 ± 0.13	0.36 ± 0.26	0.36 ± 0.13	0.,36 ± 0.17	0.36 ± 0.13	0.36 ± 0.12	0.36 ± 0.13	0.36 ± 0.14	0.36 ± 0.13

*SD* standard deviation

## Discussion

Our results have demonstrated the reproducibility of eight field tests to measure the maximal voluntary isometric muscle endurance with adapted postural positions and they have enabled the creation of predictive equations to calculate maximal theoretical voluntary isometric muscle endurance in a healthy population based on its anthropometric characteristics (age, weight, height, and gender).

The objective of the first phase of this study was to evaluate the relative and absolute reproducibility as well as the sensibility of muscle tests with modified postures for a healthy adult population, insofar as Ito et al. demonstrated that the original postures could induce pain or hyperlordosis [14]. Our results showed that all maximal voluntary isometric endurance tests present good levels of reproducibility. Indeed, for each test, the ICC was greater than 0.9, the CV less than 12% and the SEM less than 6%. The SEM of our tests was similar or lower to those found in the literature on other field tests [12, 36]. Our ICC values were higher than those obtained in other studies [12, 14, 17], but this is classic in studies using field tests to obtain an ICC > 0.81 [36, 12, 14]. The between-subject variability makes it difficult to compare the ICC between studies [26]. However, the higher ICC values in our study could be due to the fact that the population used for the reproducibility was heterogeneous, including both males and females of different ages. Nevertheless, the good to excellent absolute intra- and inter-assessor reproducibility measured by the coefficient of variation confirms our good level of reproducibility.

The sensitivity was measured by established and by analyzing for each test, whether the muscle performance is dependent on the age of the subjects. Our results showed that the observed values of the area under the ROC curves were greater than 0.7, demonstrating an influence of age on the muscle performance, which indicates young adults must have higher voluntary maximal isometric endurance than older adults. It is generally recognized that muscle endurance and strength decrease with aging [37, 38] and our study results on age-specific muscle maximal voluntary isometric endurance support the validity of our model.

The objective of the second phase of this study was to determine predictive equations of the muscle performance, taking into account the anthropometric criteria of the subjects. In the literature, the maximal voluntary isometric muscle endurance performance of field tests is often analyzed between gender and age, while height and weight are little discussed. Among all the anthropometric criteria, only the influence of age has been well established [11, 39] because of the well-known decline in muscle mass and muscle quality [40]. It is also accepted that an increase in weight has a negative influence on the performance of maximal voluntary isometric muscle endurance, but this variable is not discussed in the studies by Strand et al. and Latikka et al., which used isometric fields tests because the authors argued a weak relationship between weight and holding time during the isometric test [41, 39]. The influence of the other anthropometric criteria—gender and height—is still debated. Let us take the factor gender: one study demonstrated an absence of influence of gender on the muscle performance certain isometric tests [12], whereas other studies highlighted the effect of gender in favor of males [17, 41], or, conversely, in favor of females [42]. One hypothesis which could explain the sex-specific difference could be a difference in the muscle fiber-type distribution, muscle fiber cross-sectional area, capillary supply, oxidative and glycolytic capacity, citrate synthase, 3-hydroxyacyl-CoA-dehydrogenase, and lactate-dehydrogenase enzyme activities between men and women, but an absence of difference of these criteria between non-active adult men and women has been demonstrated [43]. With current knowledge, it is still impossible to generalize regarding the influence of gender on maximal voluntary isometric muscle endurance performance. With regard to the height factor, the literature demonstrates its influence on the holding time [39, 41], but the results of the studies are contradictory: either the holding time is better [39] or it is reduced [41] in maximal voluntary isometric muscle endurance measured with field tests. These differences could be explained by a different methodology, including athletic and non-athletic subjects [41], a group composed only of males with different age [39], or even a small number of subjects [12, 17]. In our study, the number of subjects used to analyze the influence of anthropometric parameters on the muscle performance was higher and more level-headed than the literature studies [12, 14, 17], and the profile used was the same for each group, namely, non-athletic Caucasian healthy adult subjects. Thus, our findings support that height, weight, and age are determining factors in predicting performance of maximal voluntary isometric endurance tests, unlike gender, which does not influence the performance.

Two limitations would be considered for the interpretation of these data. Our study was carried out in a French Caucasian population; thus, as explained by Hogrel et al. (2007) [44], our results could present a few variations depending on the race of the subjects (African, Asian) because of morphologic, anatomic, and cultural differences. In addition, the age bracket of the participants included in this study was from 20 to 59 years old. Thus, our predictive equations and the muscle agonist/antagonist ratios may be not be adapted for subjects who are younger or older than our participants.

## Conclusion

Our study has permitted the validation of the reproducibility of field tests and the creation of predictive equations to calculate maximal voluntary isometric muscle endurance in a healthy population according to its anthropometric characteristics, namely, age, weight, height, and gender. The creation of these normative values responds to the need to be able to assess muscle function to identify weakness or muscle imbalance in order to establish personalized training programs in the fields of prevention, sport, or rehabilitation [45].

## Practical Applications

These normative equations permit the determination of the weakness of a muscle, a muscle group, or a muscle imbalance according to the anthropometric data of the subject. The assessor simply has to complete the prediction equations with the anthropometric data of the subject (Tables 8 and 9) to obtain the theoretical value which would need to be reached at the target test. If the result is below the maximum theoretical value or if a muscle imbalance exists, physical activity management is required in the field of prevention and/or rehabilitation (risk of falling, difficulty climbing stairs, low back pain, etc.) or sport (muscle injuries risks). In addition, the predictive equations can be integrated into software or a mobile application to allow subjects to self-evaluate and benefit from an individualized program from a distance.

## Supplementary information

**Additional file 1**.

## Data Availability

The datasets generated and/or analyzed during the current study are available from the corresponding author on reasonable request.

## Abbreviations

ICC: Intraclass correlation coefficient
SEM: Standard error of the measurement
CI: Confidence interval
LOA: Limit of agreement
SEM%: Percentage of standard error of the measurement
CV: Coefficient of variation
CV%: Percentage of coefficient of variation
ROC: Receiver operating characteristic
AUC: Area under the curve
