# Supplementary material for: Isometric Strength Database for Muscle Maximal Voluntary Endurance Field Tests: Normative Data
Source: Sports Med Open. 2021 Jul 12;7:47. doi: 10.1186/s40798-021-00338-2 (PMC8273050; doi:10.1186/s40798-021-00338-2)
Supplement: Supplementary file 1 — Additional file 1. [file 40798_2021_338_MOESM1_ESM.docx]

1. Appendix 1. General instructions

***Pretest cueing for (1) trunk flexor muscles test; (2) quadratus lumborum muscles test; (3) quadriceps muscles test; (4) hamstring muscles test; and (5) back muscles test***

- To ensure the subject is able to cue the transversus abdominus (T-A), instruct them to pull their “navel to spine.”
- Monitor to ensure the subject is able to properly cue T-A.

***Subject instruction for all tests***

- One practice trial allowed to ensure the subject is able to assume position only (i.e., not held).
- Subject need breathe normally (do not block breathing).
- Subject keeps the position as long as possible.

Note: *The aim of these assessments was to determine prediction equations for each test. Therefore, we have not determined a maximum holding time for these tests, so the subjects had to maintain the position as long as possible.*

1. Appendix 2. Instructions for trunk flexor muscles test

***Starting position***

- Lying on back, the soles of feet on the ground with knees bent 90°.
- Feet and legs are joined.
- The low back is in contact with the ground.
- Hands are placed on the thighs.

***Subject instructions***

- Beforehand, perform a pelvis retroversion by pushing the low back against the ground. Instruct subject to cue T-A by drawing navel to spine.
- In this position, touches the knees while take off the shoulder blades and winding the back.
- During the test, keep the low back in contact with the ground and the pelvis in retroversion.

***Termination criteria***

- Subject terminates the test
- Subject no longer maintains the pelvis retroversion.
- Subject’s shoulder blades go down to the ground.
- Subject’s hands are not placed on their knees.
- Subject unable to properly cue T-A.

1. Appendix 3. Instructions for trunk extensor muscles test

***Starting position***

- Lying on belly, arms along the body, a small pillow placed under the lower abdomen to decrease the lumbar lordosis.
- Tiptoes and knees in contact with the ground.

***Subject instructions***

- In this position, get the chest off the ground and keep the chin on the chest, looking toward the ground.
- Maintain maximum flexion of the spine, with pelvic stabilization through gluteal muscle contraction.
- During the test, feet and knees should remain in contact with the ground.

***Termination criteria***

- Subject terminates the test.
- Feet or knees leave the ground.
- The subject’s chest touches the ground.
- The subject’s head rises and the chin is detached from the chest.

1. Appendix 4. Instructions for quadratus lumborum muscles test

***Starting position***

- Lying on side with legs extended, top foot placed over lower foot on the mat for support.
- Torso supported off the mat by the arm with elbow placed directly below shoulder.
- Once in this position, bend the knee from the leg to the ground backwards while maintaining alignment of both thighs.

***Subject instructions***

- Instruct patient to cue T-A by drawing navel to spine.
- Instruct the subject to lift hips off the mat to maintain a straight line over their body length so they are supporting themself on one elbow and their feet only.
- To help protect the shoulder girdle it will be necessary to instruct the subject to draw their shoulder toward their ipsilateral hip.
- Uninvolved arm is placed by side.
- Repeat on the other side.

***Termination criteria***

- Subject terminates the test.
- Subject’s hips return to the ground.
- Subject unable to assume position in practice trial.
- Subject unable to properly cue T-A.

1. Appendix 5. Instructions for quadriceps muscles test

***Starting position***

- Stand comfortably with feet shoulder width apart and about 2 feet from the wall, with back against a smooth vertical wall.
- Perform a pelvis retroversion by pushing the low back against the wall. Instruct subject to cue T-A by drawing navel to spine.

***Subject instructions***

- Slowly slide the back down the wall to assume a position with both knees and hips at a 90° angle.
- Move the feet a distance from the wall if required.
- The feet are flat on the ground, the back needs to be kept against the wall with a pelvis retroversion, and the knees and hips are at right angles.
- The knees should be directly above ankles (rather than over toes) and the thighs parallel to the ground.

***Termination criteria***

- Subject terminates the test.
- Modification of knee and hip angles.
- The thighs are no longer parallel to the ground.
- Subject no longer maintains the pelvis retroversion.
- Subject unable to assume position in practice trial.
- Subject unable to properly cue T-A.

1. Appendix 6. Instructions for hamstring muscles test

***Starting position***

- Lying on back, feet on the ground with both knees bent at a 90° angle.
- Place feet in heel support.
- The feet and legs are glued together.
- Perform a pelvis retroversion by pushing the low back against the ground, then place a piece of paper between knees. Instruct subject to cue T-A by drawing navel to spine.

***Subject instructions***

- In this position, press the heels into the ground to curl the pelvis and lift the buttocks of the ground.
- The buttocks are lifted a few inches.
- During this test, keep legs glued together and low back on the ground with a pelvis retroversion.

***Termination criteria***

- Subject terminates the test.
- The buttocks are returned to the ground.
- The piece of paper falls, meaning that the legs have separated.
- The low back leaves the ground.
- Subject unable to assume position in practice trial.
- Subject unable to properly cue T-A.

1. Appendix 7. Instructions for back muscles test

***Starting position***

- Sitting on the ground, the back and head leaning against a wall, the buttocks are spaced from the wall by a distance of an open hand.
- Bend both knees at a 90° angle and perform a pelvis retroversion.
- Make a double chin by holding the head against the wall while self-stretching.
- Place the arms against the wall and line them up shoulder-high.
- Elbows and fingers against the wall with elbows bent at a 90° angle.

***Subject instructions***

- Keeping fingers and elbows against the wall, slide elbows down to obtain an angle of about 45° between arms and the trunk.
- Keep elbows keep at a 90° angle.
- Keep a pelvis retroversion.

***Termination criteria***

- Subject terminates the test.
- Take fingers, elbows, or head off the wall.
- Subject no longer maintains the pelvis retroversion.
- Subject unable to assume position in practice trial.
- Subject unable to properly cue T-A.

1. Appendix 8. Instructions for chest muscles test

***Starting position***

- On all fours, place the hands on the ground so as to be more spread than the width of the shoulders.
- Arms are stretched.
- Knees and feet are glued and in contact with the ground.
- Keep the chin on the chest, looking toward the ground.

***Subject instructions***

- In this position, move the chest forward and bend the arms to bring the chest to the ground between both hands.
- Lower until elbows are aligned with shoulders with elbows bent at 90° angle.
- During the test, keep the chin on the chest, looking toward the ground.

***Termination criteria***

- Subject terminates the test.
- Subject lifts their head.
- Elbows are no longer aligned with shoulders.
